# History of Pica, Obesity, and Their Associations with Anemia in Pregnancy: A Community-Based Cross-Sectional Study

**DOI:** 10.3390/life13112220

**Published:** 2023-11-17

**Authors:** Suhaila A. Ali, Ahmed A. Hassan, Ishag Adam

**Affiliations:** 1 Department of Family and Community Medicine, Faculty of Medicine, Jazan University, Jazan 45142, Saudi Arabia; suali@jazanu.edu.sa; 2 Faculty of Medicine, University of Khartoum, Khartoum P.O. Box 102, Sudan; aa801181@gmail.com; 3 Department of Obstetrics and Gynecology, Unaizah College of Medicine and Medical Sciences, Qassim University, Unaizah 51911, Saudi Arabia

**Keywords:** anemia, hemoglobin, pica, obesity, age, body mass index, Sudan

## Abstract

Anemia in pregnancy represents a major global health problem, and progress is insufficient to meet the World Health Assembly’s global nutrition target of halving anemia prevalence by 2030. We assessed the prevalence and factors associated with anemia among pregnant women in northern Sudan. This community-based cross-sectional study was conducted at Almatamah, River Nile State, Sudan. Sociodemographic and obstetric data were collected using a questionnaire. Hemoglobin (Hb) was measured using an automated hematology analyzer. Multivariate and simple linear regression analyses were performed. A total of 586 pregnant women were enrolled. Their median (interquartile range, IQR) age was 25 (21–30) years. The median (IQR) of body mass index (BMI) was 26.67 (24.34–30.04) kg/m^2^. The median (IQR) for Hb was 11.0 (10.0–11.9) g/dL. Of the 586 women, 271 (46.2%) had anemia (Hb: <11 gm/dL). In multivariate analysis, increasing BMI and obesity were significantly associated with decreased odds ratios (ORs) of anemia, while pica was associated with increased ORs of anemia. In conclusion, anemia in pregnant women is a major public health problem, as approximately half of pregnant women in northern Sudan have anemia. Increasing BMI and obesity were associated with a lower risk for anemia. More efforts are needed to improve the maternal nutritional status for good pregnancy outcomes.

## 1. Introduction

According to the World Health Organization (WHO), pregnant women are among the most vulnerable population groups. In 2019, 32 million pregnant women aged 15–49 years were affected by anemia, with the most affected WHO regions being Africa and South-East Asia [1]. At global, regional, and national levels, anemia in pregnancy represents a major health problem, and the progress on anemia in pregnancy is insufficient to meet the World Health Assembly’s (WHA) global nutrition target to halve anemia prevalence by 2030 [2]. Lack of progress was clear in Africa, where the highest prevalence (41.7%) of anemia in pregnancy existed [3]. For example, a high prevalence of anemia in pregnancy was reported in Sudan (53.0%) [4], Ethiopia 46.2% [5], Nigeria (56.3%) [6], and Tanzania 83.5% [7]. The WHO defined anemia during pregnancy as a hemoglobin (Hb) of <11 g/dL [8].

In African countries, including Sudan, anemia in pregnancy is associated with poor maternal and perinatal outcomes, including stillbirth [9], preterm birth [9], low birth weight [9], malaria [10], postpartum hemorrhage [9], maternal blood transfusions [9], placental abruption [9], and maternal intensive care unit admission [9]. Several factors, such as low maternal education [11], low socioeconomic status [12], low body mass index (BMI) [11], urban residency [11], parity [13], not taking iron and folic acid supplements [5,14], pica [15,16], and malaria [10] are associated with anemia in pregnancy. Obesity during pregnancy is a major health problem, even in Sudan [17,18,19]. Obesity is associated with several maternal and perinatal outcomes, including maternal anemia and cesarean section [17,18,19]. While some studies showed that obesity and increasing BMI were associated with increased risk of anemia [18], others showed that obesity decreased the risk for anemia [13], and still others showed no association [14].

The WHO recommends that every pregnant woman take daily oral iron and folic acid supplements (30 mg to 60 mg of elemental iron and 0.4 mg of folic acid) to prevent maternal anemia, puerperal sepsis, low birth weight (LBW), and preterm birth [20]. However, noncompliance with WHO recommendations regarding iron and folic acid supplements is common in African countries [5].

In Sudan, different studies have shown a high prevalence of anemia in pregnancy and its complications in different regions [4,10,14]. However, little published data are available regarding anemia in pregnancy in Northern Sudan, especially in the Almatamah Locality, River Nile State [21]. Therefore, having such data from understudied areas is crucial in mapping anemia in pregnancy for effective health interventional programs to improve positive pregnancy outcomes. Stevens et al. called for a better understanding of the context-specific causes of anemia, including anemia in pregnancy, and quality implementation of effective multi-sectoral actions to address these causes [2]. The current study aimed to assess the prevalence and factors associated with anemia among pregnant women in Almatamah Locality, River Nile State, northern Sudan.

## 2. Materials and Methods

### 2.1. Study Design and Setting

A community-based cross-sectional study was conducted from July to September 2022 at Wad Hamid, Almatamah Locality, River Nile State, northern Sudan. The River Nile State is one of 18 states in Sudan. The total estimated population of this state was 1,120,000, according to the 2008 census [22]. Seven localities (the lowest administrative units in Sudan) are present in River Nile State. The Wad Hamid district borders Khartoum State and is approximately 120 km from the city of Khartoum, the capital of Sudan.

Initially, among the seven localities, Almatamah Locality was chosen as it was an understudied locality [21]. From the three districts of Almatamah Locality, one district was chosen randomly (Wad Hamid). After obtaining the entire villages’ list from the local authority, six villages were chosen randomly from the Wad Hamid district using systematic sampling. Every pregnant woman in those six villages was invited to participate in the study. A total of 90 to 110 households from each village was selected on the basis of population density to obtain the desired sample size (n = 586). As our randomization was based on village selection, every pregnant woman in the six randomly selected villages was eligible to participate. After signing an informed consent form, all pregnant women were enrolled.

### 2.2. Outcome Definitions

The main outcome measure was anemia. According to the WHO, anemia in pregnancy was defined as an Hb concentration of <11 g/dL [8]. Furthermore, anemia was categorized into three groups: mild anemia (Hb of 11–11.9 g/dL in women and 11–12.9 g/dL in men), moderate anemia (Hb of 8–10.9 g/dL), and severe anemia (Hb of < 8 g/dL) [8]. However, in the current study, any woman with a Hb concentration of <11 g/dL was defined as an anemic woman, and others with a Hb concentration of ≥11 g/dL as non-anemic women.

Furthermore, based on the mean corpuscular volume (MCV), anemia was sub-classified into microcytic anemia (MCV < 80 fl), normocytic anemia (MCV 80 to 100 fl), and macrocytic anemia (MCV > 100 fl).

### 2.3. Study Participants

During the study period, every pregnant woman with a singleton pregnancy in the selected villages was approached by trained female medical officers; therefore, any woman in those villages was eligible to participate in the study. Any pregnant woman who refused to give informed consent or who had multiple pregnancies, vaginal bleeding in the index pregnancy, or systemic diseases, such as diabetes mellitus, hypertension, thyroid disease, hemolytic disease, or intrauterine fetal demise, was excluded from the study. The purpose of the study and the ethical issues were explained to the eligible participants by the medical officers.

### 2.4. Data Collection

In this study, the Strengthening the Reporting of Observational Studies in Epidemiology (STROBE) guidelines were strictly followed [23]. A pretested questionnaire was used to collect the data. The questionnaire had been tested among a small sample group (ten women), and all necessary corrections were performed before data collection. The questionnaire was based on previous similar studies from similar contexts in eastern and central Sudan [14,15]. The questionnaire included sociodemographic data, such as maternal age in years, maternal education (<secondary or ≥secondary), and occupation (employed or housewife), as well as obstetrical information, such as gravidity, history of cesarean section, and history of abortion. In this study, the authors followed the definition of symptoms of pica as the “persistent desire to eat uncooked food or non-food substances for at least one month”, which was recently used in Uganda [24].

### 2.5. Anthropometric Measurements

Each woman’s weight was measured in kilograms (kg) using well-calibrated scales and adjusted to zero before each measurement according to the standard procedure. The woman stood with minimal movement, with hands by their sides and shoes and excess clothing removed. The woman’s height was measured in centimeters (later converted into meters) after standing straight with her back against a wall and her feet together. BMI was computed as the weight in kg divided by the square of the height in meters (kg/m^2^) [25]. Furthermore, BMI was grouped according to the WHO classification as underweight (<18.5 kg/m^2^), normal weight (18.5–24.9 kg/m^2^), overweight (25.0–29.9 kg/m^2^), or obese (≥30.0 kg/m^2^) [25].

### 2.6. Blood Analysis

Each woman was requested to give 3 mL of blood for Hb and blood group analysis. These samples were used as part of a complete blood count. An automated hematology analyzer (Sysmex KX-21, Kobe, Japan) was used to measure Hb levels and MCV, as described in our previous work [26]. Maternal blood group typing was performed using a manual agglutination method (blood groups). From the 3 mL blood, one drop was put into each of four round-bottom tubes, then a drop of monoclonal anti-A, anti-B, and monoclonal/polyclonal anti-D was put into each tube, mixed well over an area of 2.5 cm, and rocked gently back and forth. The results were interpreted as follows: for ABO blood groups, the agglutination was recorded immediately, as described in previous studies [27,28].

Both thick and thin blood films were stained with Giemsa, examined using 100 oil immersion fields, and double-checked blindly. Three women were diagnosed with malaria; they were anemic, and we excluded them from the analysis.

### 2.7. Sample Size Calculation

A sample of 586 pregnant women was calculated using Epi-Info [29]. Based on a previous study in Sudan, we assumed a prevalence of anemia of 50% among pregnant women [4]. Thereafter, depending on the previous reports in eastern Sudan [15], we assumed that 42.0% of the women with anemia would have a history of pica, and 30% of women without anemia would have a history of pica. To have a sufficient sample size, a prevalence of anemia of 50% among pregnant women was used in this study. As this was a cross-sectional and not a longitudinal study, we assumed no potential losses that need to be estimated in the sample size calculation. This sample has 80% power, with a precision of 5%.

### 2.8. Statistical Analysis

For this study analysis, the IBM Statistical Package for the Social Sciences^®^ (SPSS^®^) for Windows, version 22.0 (SPSS Inc., New York, NY, USA) was used to analyze the data. The proportions were expressed as frequencies (%). The Shapiro–Wilk test for determining the normality of continuous data (maternal age, gravidity, BMI, and Hb level) revealed a non-normal distribution, and the data were expressed as the median (interquartile range, IQR). A Student *t*-test was used to compare normally distributed data between two groups. Univariate analysis was performed for anemia as the dependent variable and sociodemographic variables (maternal age, maternal educational level, and maternal occupational status), anthropometric measurement BMI, BMI groups (entered one by one in the analysis model), and obstetrical data (gravidity, history of cesarean section, history of abortion, and blood group) as independent variables. A multivariate analysis was also performed, including all variables with a *p*-value of <0.2 to control for confounding variables. Furthermore, simple linear regression was performed to explore the association between the Hb (gm/dL) level and BMI (kg/m^2^). Adjusted odds ratios (AORs), 95% confidence intervals (CIs) coefficients, and standard error were calculated. A two-sided *p*-value of <0.05 was considered statistically significant.

## 3. Results

A total of 586 pregnant women were analyzed in this study. The median (IQR) of their age and gravidity were 25 (21–30) years, and 3 (1–5), respectively. Of the total, 394 (67.4%) women had four or more antenatal care visits, and the remaining 191 (32.6%) had fewer than four. Of the total 586 women, 418 (71.3%) were educated (secondary school level or higher), and 168 (28.7%) were uneducated (less than secondary school level). The majority of the women were housewives (548; 93.5%). In total, 120 (20.5%) women and 94 (16%) women had a history of cesarean delivery and abortion, respectively. Of the 586 women, 343 (58.5) used iron and folic acid during pregnancy. Of the total 586 women, 221 (37.7%) women had a history of pica. Of the total 586, 360 (61.4%) were blood group O, and the remaining 226 (38.6%) were other than blood group O. The median (IQR) of BMI was 26.67 (24.34–30.04) kg/m^2^. A total of 4 (0.7%), 180 (20.7%), 255 (43.5%), and 147 (25.1%) of the 586 pregnant women were underweight, normal weight, overweight, and obese, respectively (Table 1).

The median (IQR) of Hb was 11.0 (10.0–11.9) g/dL, with a range from 5.5 to 14.0 g/dL. Of the total 586 women, 271 (46.2%) had anemia (Hb: <11 gm/dL); 229 (39.1%) had mild anemia (Hb: 9.0–10.9 gm/dL); 36 (6.1%) had moderate anemia (Hb: 7.0–8.9 gm/dL); and 6 (1.0%) had severe anemia (Hb: <7 gm/dL). The mean (SD) of the total MCV (fl) was 87.2 (7.3), with 95% CI 85.7–87.5, ranging from 66 to 109 fl. The mean (SD) of the MCV was not different in women with and without anemia: 85.9 (6.5) fl versus 88.4 (6.9) fl, *p* = 0.311. Of 586, 260 (44.3%) women had microcytic anemia (MCV < 80 fl). The median (IQR) BMI was higher in women with anemia (27.69 kg/m^2^; 24.97–30.82) than without anemia (25.96 kg/m^2^; 23.73–29.17). Univariate analysis revealed that BMI, obesity, and pica were significantly associated with anemia. Multivariate analysis showed that maternal age, maternal education, history of abortion, underweight, and overweight were not associated with anemia, whereas increasing BMI (AOR 0.94, 95% CI 0.91–0.98, *p* = 0.001) and obesity (AOR 0.50, 95% CI 0.32–0.81, *p* = 0.004) were significantly associated with decreased ORs for anemia, and that history of pica (AOR 1.76, 95% CI 1.24–2.48, *p* = 0.001) was associated with increased ORs for anemia (Table 2).

**Figure 1:**
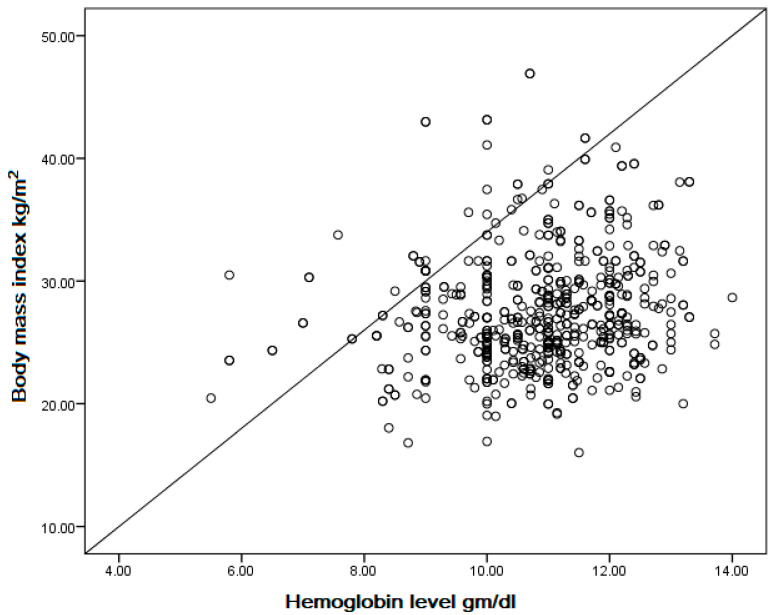
Correlation between body mass index (kg/m^2^) and hemoglobin level (gm/dL) among pregnant women in Almatamah, River Nile State, northern Sudan (n = 586) (coefficient = 0.048, *p* < 0.001).

In simple linear regression, there is a positive correlation between body mass index (kg/m^2^) and hemoglobin level (gm/dL) among pregnant women (coefficient = 0.048, *p* < 0.001) (Figure 1).

## 4. Discussion

The main findings of the present study were the identification of a high prevalence of anemia (46.2%) and its associated factors among pregnant women in northern Sudan. The main factors associated with anemia were a history of pica and BMI. These findings indicate that anemia in pregnancy is a public health problem in Sudan. Previous studies showed similar results in the White Nile State, Sudan (42.3%) [14], eastern Sudan (62.6%) [15], and in a Sudan household survey (36%) [10]. A meta-analysis that included 16 studies and enrolled 15,688 pregnant Sudanese women reported a pooled prevalence of anemia of 53.0% [4]. Several other studies conducted in Africa revealed a high prevalence of anemia in pregnancy in Ethiopia (46.2%) [5], Nigeria (56.3%) [6], Somali (64.8%) [9], and Tanzania (83.5%) [7]. By contrast, a low prevalence of anemia in pregnancy (26.9%) was reported in Saudi Arabia [13].

Clearly, substantial variations in the prevalence of anemia exist among countries and even in the same country between regions [2]. For example, at the country level (Sudan), anemia in pregnancy ranged from 36% in the Sudan household survey [10] to 62.6% in eastern Sudan [15]. At the global level, according to the meta-analysis results, the overall prevalence of anemia in pregnant women was 36.8%, but the highest prevalence of anemia in pregnant women was in Africa (41.7%) [3]. Furthermore, the high prevalence of anemia in pregnancy in low-income countries compared to high-income countries was attributed to socioeconomics and health factors [3]. The health factors responsible for these differences might include malnutrition (underweight), exposure to malaria, and a high HIV prevalence, as these factors have strong associations with maternal anemia [11]. This indicates that each country needs to estimate its prevalence and propose suitable interventions based on local findings to prevent anemia in pregnancy [2]. In Sudan, the variation in the prevalence of anemia in pregnancy could be explained by malaria, which is endemic in certain regions [4,10]. As mentioned above, three women in our cohort had malaria and anemia. Pregnant Sudanese women who had malaria infection during pregnancy were 1.94 times (OR 1.94) more likely to develop anemia than women who had no malaria infection [4].

This study revealed that 37.7% of pregnant women had a history of pica and had a 1.76 times higher risk of anemia. Several studies from different countries, including Sudan, reported the impacts of pica on anemia in pregnancy [15,16]. A previous study in eastern Sudan among 466 pregnant women showed that pica (AOR 1.6) was associated with an increased risk of anemia [15]. A previous study in central Sudan among 292 pregnant women showed that 119 (40.8%) women had anemia, and while maternal age, parity, education, occupation, and BMI were not associated with anemia, pica was associated with anemia (AOR = 1.7) [16]. Conversely, a previous Sudanese study conducted in Khartoum (an urban community) showed no association between pica and maternal anemia [30]. The association between pica practice and anemia could have several possible explanations. One of them is the influence of pica on nutritional status and the stress and anxiety of pregnant women [31]. An updated global meta-analysis showed a pica prevalence of 38% in pregnant women and that rural women were at a higher risk of pica [32]. The influence of pica on Hb level, especially as observed in rural vs. urban regions, could be due to the types of pica (mud, ice, etc.). A further in-depth study to investigate the influence of each pica type on Hb levels is desirable. For example, pica might increase the exposure of pregnant women to helminth infections and micronutrient deficiency, both of which can result in anemia [24]. Also, pica could reduce iron absorption [33]. Therefore, patients with iron deficiency anemia need a holistic approach, i.e., to take a full history of practicing pica aiming for early diagnosis, treatment, and prevention of complications [34].

The current study showed that pregnant women with increasing BMI and obesity were less likely to have anemia. There was a positive correlation between BMI (kg/m^2^) and Hb level (gm/dL) among pregnant women (coefficient = 0.048, *p* < 0.001). In particular, an increase of one unit in BMI (I unit BMI means 1 kg/m^2^) decreased anemia by 6% (AOR = 0.94, 95% CI 0.91–0.98), while a determination of obesity decreased anemia by 50% (AOR 0.50, 95% CI 0.32–0.81). These findings are in agreement with previous studies conducted in Sudan and Saudi Arabia, which showed similar results [13,35]. In central Sudan, a previous study among 423 pregnant women showed that anemia is infrequent in overweight and obese pregnant women despite their high risk of iron deficiency (ID) [35]. A cross-sectional study in Saudi Arabia on 334 pregnant women revealed that increasing BMI (AOR 0.93, 95% CI 0.89–0.97) and obesity (AOR 0.31, 95% CI 0.16–0.61) were associated with decreased odds of maternal anemia [13]. Launbo et al., in their review, also found a coexistence of obesity and anemia during pregnancy [18]. Other studies have shown no association between BMI and anemia among pregnant women in central Sudan [14,16] or Khartoum, Sudan [30]. These different influences of BMI on anemia in pregnancy could be explained by different sociodemographic characteristics, differences in the prevalence of anemia and obesity, and different inclusion and exclusion criteria used in the studies. Obesity could increase anemia via inflammation, as adiposity has been extensively linked to inflammation [36]. Conversely, when increased BMI is related to increases in muscular bulk, obesity could decrease anemia because of good nutrition [36].

The findings of the present study showed no association between age or gravidity and maternal anemia. Similarly, our previous studies, which included a systematic review and meta-analysis, showed no association between age or gravidity and maternal anemia in Sudan [4,16,30].

The present study also did not reveal any association between maternal education, occupation, or history of abortion and maternal anemia. In Sudan, our cross-sectional study, which included 208 women, showed that maternal anemia was not associated with age, education, parity, history of abortion, or antenatal care level [14]. By contrast, a previous study in Khartoum, Sudan, showed that pregnant women with low education levels [30] had a 2.3 times higher risk of anemia. These variations in the associated factors could have many explanations, such as the impact of education on antenatal care due to the quality of the education provided to each parent [37] and the effectiveness of education in empowering women financially to make decisions regarding their own health, including the provision of good antenatal care. This variation in the influence of maternal education and occupation could also be explained by the low quality of the education provided to females [37], especially in rural settings, as well as the low possibility that even educated women can find employment (i.e., in this study, only 6.5% of the women were employed) and other impacts due to socioeconomic status. In Africa, low maternal education [11] and low socioeconomic status [12] are well-known risk factors for anemia in pregnancy.

This study showed no association between antenatal care visits and anemia. Our previous studies in central Sudan and Saudi Arabia also showed similar results [13,14]; however, other researchers have identified antenatal care as a preventive factor of anemia [38,39]. The lack of association in our context might reflect the poor quality of the antenatal care services provided (i.e., the focus should be on the quality of the antenatal care services rather than on the number of visits). A point worth mentioning is that both low antenatal coverage and low quality of antenatal care have been reported in Sudan [40].

In the present study, not taking iron and folic acid supplements was not associated with maternal anemia. Other studies from various countries, including Sudan, have shown similar results [15,30]. By contrast, other studies, including our previous studies in central Sudan [14] and Ethiopia [5], found an association between not taking iron and folic acid supplements and maternal anemia. These contradictory results could be explained by differences in compliance with iron and folic acid supplements (i.e., the duration and commitment of women to take iron and folic acid supplements as prescribed by health professionals). In the current study, microcytic anemia was more common (44.3%). This is similar to our previous study that revealed a high prevalence of iron deficiency anemia among pregnant women in Sudan [35].

A woman’s blood type was not associated with anemia; however, resistance to anemia has been reported in individuals with blood group O compared to individuals with other blood group types [41]. Blood group O resistance to anemia could be explained by several reasons. Of them, Rowe et al. [42] reported that blood group O may confer resistance to severe falciparum malaria via the mechanism of reduced rosetting. Our previous studies in Sudan showed a strong association between anemia and malaria [15,43]. Furthermore, there is an ongoing debate regarding blood type diet, i.e., each blood group is associated with certain food types [44].

The present study showed no association between a history of abortion and anemia. However, in South-East Ethiopia, anemia was less likely in pregnant women with no history of abortion than in pregnant women who had a history of abortion (AOR 0.4) [5]. The differences in the results between studies could be due to the number of abortions rather than the absence or presence of abortion.

To the best of our knowledge, this is the first study to investigate anemia in pregnancy in this understudied area (Almatamah). The findings presented here provide valuable data for decision makers to create interventional programs that include nutritional and pica awareness to improve pregnancy outcomes. Despite these strengths, this study has some limitations that must be acknowledged and overcome in future studies. First, the current study is a quantitative study; therefore, other studies using mixed methods (both quantitative and qualitative) for data collection will be of paramount importance for exploring anemia and its related factors, including nutritional factors and pica practice. Second, the nature of the study (i.e., its cross-sectional approach) creates difficulties when trying to establish causality between anemia and the investigated factors (especially pica and BMI). Further longitudinal studies with more patients and with public involvement and engagement will provide real added value to estimates of the impact of anemia on maternal and perinatal health. For example, our previous studies revealed a negative impact of anemia on maternal and perinatal outcomes in Sudan [4,10,14]. Although MCV was used to sub-classify anemia into microcytic, normocytic, and macrocytic in the present study, unlike other studies, serum ferritin levels were not measured [35], and dietary intake was not collected [6]. Knowledge of this information is crucial for developing interventions to improve maternal and perinatal health.

## 5. Conclusions

Anemia among pregnant women is a major public health problem in Sudan, as approximately half of the pregnant women in northern Sudan have anemia. Our results showed that increasing BMI and obesity were associated with a lower risk of anemia. More efforts are needed to improve the maternal nutritional status for good pregnancy outcomes.

## Figures and Tables

**Table 1:** Univariate analysis of factors associated with anemia in pregnant women in Almatamah, River Nile State, northern Sudan (n = 586).

Variables		Total (n = 586)	Anemic Women (Hemoglobin: <11 gm/dL) n = 271 (46.2%)	Non-Anemic Women (Hemoglobin: ≥11 gm/dL) n = 315 (53.8%)	Odds Ratio (95% Confidence Interval)	*p*-Value
		Median (interquartile range)				
Maternal age, years		25.0 (21–30)	25.0 (21.0–29)	25.0 (21.0–30)	0.98 (0.95–1.01)	0.086
Gravidity		3 (1–5)	3 (1–4)	3 (1–5)	0.96 (0.90–1.03)	0.284
Body mass index, kg/m^2^		26.67 (24.34–30.04)	25.96 (23.73–29.17)	27.69 (24.97–30.82)	0.94 (0.91–0.97)	0.001
		n (%)	n (%)	n (%)		*p*-value
Antenatal care	≥4 visit	395 (67.4)	189 (69.7)	206 (65.4)	Reference	
	<4 visit	191 (32.6)	82 (30.3)	109 (34.6)	0.82 (0.58–1.16)	0.264
Maternal education status	≥secondary	418 (71.3)	204 (75.3)	214 (67.9)	Reference	
	<secondary	168 (28.7)	67 (24.7)	101 (32.1)	0.69 (0.48–1.01)	0.051
Maternal employment status	Employed	38(6.5)	20(7.4)	18(5.7)	Reference	
	Housewife	548 (93.5)	251 (92.6)	297 (94.3)	0.76 (0.39–1.47)	0.415
History of caesarean section	No	466 (79.5)	220 (81.2)	246 (78.1)	Reference	
	Yes	120 (20.5)	51 (18.8)	69 (21.9)	0.83 (0.55–1.24)	0.356
History of abortion	No	492 (84.0)	234 (86.3)	258 (81.9)	Reference	
	Yes	94 (16.0)	37 (13.7)	57 (18.1)	0.72 (0.46–1.12)	0.145
Usage of folic iron	Yes	343 (58.5)	158 (58.3)	185 (58.7)	Reference	
	No	243 (41.5)	113 (41.7)	130 (41.3)	1.02 (0.73–1.42)	0.917
Pica	No	365 (62.3)	152 (56.1)	213 (67.6)	Reference	
	Yes	221 (37.7)	119 (43.9)	102 (32.4)	1.62 (1.17–2.29)	0.004
Body mass index groups	Normal	180 (30.7)	96 (35.4)	84 (26.7)	Reference	0.013
	Underweight	4 (0.7)	3 (1.1)	1 (0.3)	2.63 (0.27–25.72)	
	Overweight	255 (43.5)	119 (43.9)	136 (43.2)	0.77 (0.52-–1.12)	
	Obese	147 (25.1)	53 (19.6)	94 (29.8)	0.49 (0.32–77)	
Blood groups	Blood group O	360 (61.4)	167 (61.6)	122 (38.7)	Reference	
	Other than O	226 (38.6)	104 (38.4)	193 (61.3)	1.02 (0.73–1.42)	0.930

**Table 2:** Multivariable logistic regression analyses of factors associated with anemia (hemoglobin < 11 gm/dL) among pregnant women in Almatamah, River Nile State, northern Sudan (n = 586).

Variable		Adjusted Odds Ratio	95% Confidence Interval	*p*-Value
Maternal age, years		0.99	0.96–1.02	0.504
Body mass index kg/m^2^		0.94	0.91–0.98	0.001
Maternal educational status	≥secondary	Reference		
	<secondary	0.71	0.49–1.03	0.075
History of abortion	No	Reference		
	Yes	0.72	0.45–1.16	0.179
Pica eater	No (Reference)			
	Yes	1.76	1.24–2.48	0.001
Body mass index groups	Normal	Reference		
	Underweight	2.51	0.26–24.80	0.430
	Overweight	0.80	0.54–1.18	0.254
	Obese	0.50	0.32–0.81	0.004

## Data Availability

The data supporting the current study’s findings will be available upon rational request from the corresponding author.

## References

[1] World Health Organization Anaemia: Key Facts. https://www.who.int/news-room/fact-sheets/detail/anaemia#:~:text=Anaemia%20occurs%20when%20there%20isn,pregnant%20women%20and%20their%20babies.

[2] Stevens G.A., Paciorek C.J., Flores-Urrutia M.C., Borghi E., Namaste S., Wirth J.P., Suchdev P.S., Ezzati M., Rohner F., Flaxman S.R. (2022). National, regional, and global estimates of anaemia by severity in women and children for 2000–19: A pooled analysis of population-representative data. Lancet Glob. Health.

[3] Karami M., Chaleshgar M., Salari N., Akbari H., Mohammadi M. (2022). Global Prevalence of Anemia in Pregnant Women: A Comprehensive Systematic Review and Meta-Analysis. Matern. Child Health J..

[4] Adam I., Ibrahim Y., Elhardello O. (2018). Prevalence, types and determinants of anemia among pregnant women in Sudan: A systematic review and meta-analysis. BMC Hematol..

[5] Girma S., Teshome T., Worku M., Solomon T., Kehulu S., Aman R., Bonsa M., Assefa T., Gezahegn H. (2020). Anemia and associated factors among pregnant women attending antenatal care at madda walabu university goba referral hospital, bale zone, south-east ethiopia. J. Blood Med..

[6] Oyewole Oyerinde O., Nkanga E.A., Oyerinde I.E., Akintoye O., Asekun-Olarinmoye I., Alabi Q.K. (2023). Factors affecting anemia in pregnancy aomen in Ibeju-Lekki, Lagos State, Nigeria. INQUIRY J. Health Care Organ. Provis. Financ..

[7] Ngimbudzi E.B., Massawe S.N., Sunguya B.F. (2021). The Burden of Anemia in Pregnancy Among Women Attending the Antenatal Clinics in Mkuranga District, Tanzania. Front. Public Health.

[8] Ramsay M. (2010). Normal hematological changes during pregnancy and the puerperium. The Obstetric Hematology Manual.

[9] Barut A., Mohamud D.O. (2023). The association of maternal anaemia with adverse maternal and foetal outcomes in Somali women: A prospective study. BMC Women’s Health.

[10] Elmardi K.A., Adam I., Malik E.M., Abdelrahim T.A., Elhag M.S., Ibrahim A.A., Babiker M.A., Elhassan A.H., Kafy H.T., Elshafie A.T. (2020). Prevalence and determinants of anaemia in women of reproductive age in Sudan: Analysis of a cross-sectional household survey. BMC Public Health.

[11] Correa-Agudelo E., Kim H.Y., Musuka G.N., Mukandavire Z., Miller F.D.W., Tanser F., Cuadros D.F. (2021). The epidemiological landscape of anemia in women of reproductive age in sub-Saharan Africa. Sci. Rep..

[12] Abdallah F., John S.E., Hancy A., Paulo H.A., Sanga A., Noor R., Lankoande F., Chimanya K., Masumo R.M., Leyna G.H. (2022). Prevalence and factors associated with anaemia among pregnant women attending reproductive and child health clinics in Mbeya region, Tanzania. PLoS Glob. Public Health.

[13] Eltayeb R., Binsaleh N.K., Alsaif G., Ali R.M., Alyahyawi A.R., Adam I. (2023). Hemoglobin levels, anemia, and their associations with body mass index among pregnant women in Hail Maternity Hospital, Saudi Arabia: A cross-sectional study. Nutrients.

[14] Elmugabil A., Adam I. (2023). Prevalence and Associated Risk Factors for Anemia in Pregnant Women in White Nile State, Sudan: A Cross-Sectional Study. SAGE Open Nurs..

[15] Adam I., Khamis A.H., Elbashir M.I. (2005). Prevalence and risk factors for anaemia in pregnant women of eastern Sudan. Trans. R. Soc. Trop. Med. Hyg..

[16] Abdelgadir M.A., Khalid A.R., Ashmaig A.L., Ibrahim A.R.M., Ahmed A.-A.M., Adam I. (2012). Epidemiology of anaemia among pregnant women in Geizera, central Sudan. J. Obstet. Gynaecol..

[17] Chen C., Xu X., Yan Y. (2018). Estimated global overweight and obesity burden in pregnant women based on panel data model. PLoS ONE.

[18] Launbo N., Davidsen E., Granich-Armenta A., Bygbjerg I.C., Sánchez M., Ramirez-Silva I., Avila-Jimenez L., Christensen D.L., Rivera-Dommarco J.A., Cantoral A. (2022). The overlooked paradox of the coexistence of overweight/obesity and anemia during pregnancy. Nutrition.

[19] Taha Z., Hassan A.A., Wikkeling-Scott L., Papandreou D. (2019). Prevalence and associated factors of caesarean section and its impact on early initiation of breastfeeding in Abu Dhabi, United Arab Emirates. Nutrients.

[20] World Health Organization (2016). WHO Recommendations on Antenatal Care for a Positive Pregnancy Experience.

[21] Albadri F.A., Hamad M.N.M., Babiker A.M., Elgasim N., Jubara A.E.J.E., Hussein A.A. (2022). Prevalence of anemia among pregnant ladies attended to Alfadlab Hospital, River Nile State, Sudan. SAR J. Med..

[22] 5th Sudan Population and Housing Census—2008. https://microdata.worldbank.org/index.php/catalog/1014.

[23] von Elm E., Altman D.G., Egger M., Pocock S.J., Gøtzsche P.C., Vandenbroucke J.P. (2008). The Strengthening the Reporting of Observational Studies in Epidemiology (STROBE) statement: Guidelines for reporting observational studies. J. Clin. Epidemiol..

[24] Nakiyemba O., Obore S., Musaba M., Wandabwa J., Kiondo P. (2021). Covariates of pica among pregnant women attending antenatal care at Kawempe Hospital, Kampala, Uganda: A Cross-sectional study. Am. J. Trop. Med. Hyg..

[25] World Health Organization (2000). Obesity: Preventing and Managing the Global Epidemic: Report of a WHO Consultation. www.OpenEpi.com.

[26] Abdelrahman E.G., Gasim G.I., Musa I.R., Elbashir L.M., Adam I. (2012). Red blood cell distribution width and iron deficiency anemia among pregnant Sudanese women. Diagn. Pathol..

[27] Abbas A.A. (2017). Frequency of ABO and Rh D blood groups among Sudanese blood donors attending Central Blood Bank in Wad Medani, Gezira State, Sudan. Int. J. Med. Res. Prof..

[28] Adam I., Babiker S., Mohmmed A.A., Salih M.M., Prins M.H., Zaki Z.M. (2007). ABO blood group system and placental malaria in an area of unstable malaria transmission in eastern Sudan. Malar. J..

[29] Dean A.G., Sullivan K.M., Soe M.M. OpenEpi: Open Source Epidemiologic Statistics for Public Health, Version 4; Updated 6 April 2013. www.OpenEpi.com.

[30] Mubarak N., Gasim G.I., Khalafalla K.E., Ali N.I., Adam I. (2014). Helicobacter pylori, anemia, iron deficiency and thrombocytopenia among pregnant women at Khartoum, Sudan. Trans. R. Soc. Trop. Med. Hyg..

[31] Francis S., Jagadeesh N.S., Singaravelu R., Subramaniam A. (2022). The influence of pica practice on nutritional status, stress and anxiety of pregnant women. Clin. Epidemiol. Glob. Health.

[32] Sanjari S., Reza M., Soleimani M., Fakhraei A.A. (2023). Update on the Global Prevalence of Pica in Pregnant Women: A Meta-analysis. Int. J. Women’s Health Reprod. Sci..

[33] Borgna-Pignatti C., Zanella S. (2016). Pica as a manifestation of iron deficiency. Expert Rev. Hematol..

[34] Ganesan P.R., Vasauskas A.A. (2023). The association between pica and iron- deficiency anemia: A scoping review. Cureus.

[35] Abbas W., Adam I., Rayis D.A., Hassan N.G., Lutfi M.F. (2017). Higher Rate of Iron Deficiency in Obese Pregnant Sudanese Women. Open Access Maced. J. Med. Sci..

[36] El-Mallah C.A., Beyh Y.S., Obeid O.A. (2021). Iron fortification and supplementation: Fighting anemia of chronic diseases or fueling obesity?. Curr. Dev. Nutr..

[37] Gordon R., Marsto L., Rose P., Zubairi A. (2009). 12 Years of Quality Education for All Girls: A Commonwealth Perspective. https://www.educ.cam.ac.uk/centres/real/downloads/Platform%20for%20Girls/REAL%2012%20Years%20of%20Quality%20Education%20for%20All%20Girls%20FULL%2084pp.pdf.

[38] Saapiire F., Dogoli R., Mahama S. (2022). Adequacy of antenatal care services utilisation and its effect on anaemia in pregnancy. J. Nutr. Sci..

[39] Kare A.P., Gujo A.B. (2021). Anemia among pregnant women attending antenatal care clinic in Adare General Hospital, Southern Ethiopia: Prevalence and associated factors. Health Serv. Insights.

[40] Ali A.A.A., Mohammed A.A., Sulaiman M.A. (2011). Education, poor antenatal care coverage and teenage pregnancy at Kassala Hospital, Eastern Sudan. J. Public Health Epidemiol..

[41] Asim Kumar B., Kaushik M. (2013). Blood group and anemia: Exploring a new relationship. J. Public Health Epidemiol..

[42] Rowe J.A., Handel I.G., Thera M.A., Deans A.M., Lyke K.E., Koné A., Diallo D.A., Raza A., Kai O., Marsh K. (2007). Blood group O protects against severe Plasmodium falciparum malaria through the mechanism of reduced rosetting. Proc. Natl. Acad. Sci. USA.

[43] Adam I., Elhassan E.M., Haggaz A.E.D., Ali A.A.A., Adam G.K. (2011). A perspective of the epidemiology of malaria and anaemia and their impact on maternal and perinatal outcomes in Sudan. J. Infect. Dev. Ctries..

[44] Shmerling R.H. (2022). Diet Not Working? Maybe Its Not Your Type What’s the Blood Type Diet?.

